# Drug-induced aneuploidy and polyploidy is a mechanism of disease relapse in MYC/BCL2-addicted diffuse large B-cell lymphoma

**DOI:** 10.18632/oncotarget.26251

**Published:** 2018-11-13

**Authors:** Shariful Islam, Andrew L. Paek, Michael Hammer, Savithri Rangarajan, Robert Ruijtenbeek, Laurence Cooke, Eric Weterings, Daruka Mahadevan

**Affiliations:** ^1^ Cancer Biology GIDP, University of Arizona Cancer Center, Tucson, AZ, USA; ^2^ Department of Molecular and Cellular Biology, University of Arizona, Tucson, AZ, USA; ^3^ Division of Biotechnology, University of Arizona Cancer Center, Tucson, AZ, USA; ^4^ PamGene International B.V., 's-Hertogenbosch, The Netherlands; ^5^ Department of Medicine, University of Arizona Cancer Center, Tucson, AZ, USA; ^6^ Department of Radiation Oncology, University of Arizona, Tucson, AZ, USA

**Keywords:** DLBCL, aurora kinase, aneuploidy-polyploidy, TPX2, RanGAP1

## Abstract

Double-hit (DH) or double-expresser (DE) lymphomas are high-grade diffuse large B-cell lymphomas (DLBCL) that are mostly incurable with standard chemo-immunotherapy due to treatment resistance. The generation of drug-induced aneuploid/polyploid (DIAP) cells is a common effect of anti-DLBCL therapies (e.g. vincristine, doxorubicin). DIAP cells are thought to be responsible for treatment resistance, as they are capable of re-entering the cell cycle during off-therapy periods. Previously we have shown that combination of alisertib plus ibrutinib plus rituximab can partially abrogate DIAP cells and induce cell death. Here, we provide evidence that DIAP cells can re-enter the cell cycle and escape cell death during anti-DLBCL treatment. We also discuss MYC/BCL2 mediated molecular mechanism that underlie treatment resistance. We isolated aneuploid/polyploid populations of DH/DE-DLBCL cells after treatment with the aurora kinase (AK) inhibitor alisertib. Time-lapse microscopy of single polyploid cells revealed that following drug removal, a subset of these DIAP cells divide and proliferate by reductive cell divisions, including multipolar mitosis, meiosis-like nuclear fission and budding. Genomic, proteomic, and kinomic profiling demonstrated that alisertib-induced aneuploid/polyploid cells up-regulate DNA damage, DNA replication and immune evasion pathways. In addition, we identified amplified receptor tyrosine kinase and T-cell receptor signaling, as well as MYC-mediated dysregulation of the spindle assembly checkpoints *RanGAP1, TPX2 and KPNA2*. We infer that these factors contribute to treatment resistance of DIAP cells. These findings provide opportunities to develop novel DH/DE-DLBCL therapies, specifically targeting DIAP cells.

**Key Points:**

● MYC mediated upregulation of TPX2, KPNA2 and RanGAP1 dysregulate the spindle assembly checkpoint in drug-induced polyploid cells.

● Drug-induced polyploid cells re-enter the cell cycle via multipolar mitosis, fission or budding, a mechanism of disease relapse.

## INTRODUCTION

Diffuse large B-cell lymphoma (DLBCL), an aggressive form of B-cell non-Hodgkin lymphoma (B-NHL), is the most common subtype in adults (∼30%) with ∼45,000 new cases per year. Double Hit (DH) (germinal center origin) or Double Expresser (DE) (activated B-cell origin) DLBCL of comprise ∼20-25% of newly diagnosed high grade (HG) DLBCL and are defined by either translocation or over-expression of MYC and BCL2, respectively. While ∼60% of DLBCL patients have long term complete remissions with standard R-CHOP-like chemo-immunotherapy, the remaining ∼40% (which includes DH/DE- HG-DLBCL) are resistant to therapy with a 2-year overall survival of ∼20% [1], which might improve further with the approval of CAR-T therapy by the US-FDA [2]. The mechanism(s) of primary treatment resistance are not known with drugs that predominantly target the cell cycle. There is clearly an unmet medical need for the development of novel therapies to treat DH/DE-DLBCL.

MYC is a dominant oncogenic transcription factor that regulates the expression of many cell cycle kinases including aurora kinases (AK) A and B. AKs are mitotic serine/threonine protein kinases that play key regulatory roles in the mitotic phase of the eukaryotic cell cycle. MYC interacts with an anti-apoptotic network via the BCL2 family of proteins that regulates the intrinsic mitochondrial pathway of apoptosis [3]. MYC is not directly targetable but inhibiting AK with small molecule inhibitors can indirectly inhibits MYC effects in cancer cells [4–6].

Anti-DLBCL chemotherapy dosing schedules are intermittent to avoid damage to normal tissue such as the mucous membranes, intestinal lining, and the bone marrow. Intermittent dosing, however, can result in continued tumor growth during off-therapy periods and the development of treatment resistance. Multiple cell cycle kinase inhibitors are able to induce polyploidy [7–12] as do DNA-targeted (e.g. anthracyclins, etoposide) and microtubule-targeted (e.g. vinca alkaloids, taxanes) anti B-NHL chemotherapies [13]. Some polyploid cells die during treatment, but the remaining cells are speculated to undergo reductive cell divisions(s) via multipolar mitosis, meiosis-like cell division or budding, similar to what is observed in megakaryocytes during platelet release [14, 15]. Error-prone chromosome segregations of polyploid cells may result in aneuploidy [16]. The surviving population of aneuploid cells is surmised as drug resistant and a mechanism of therapy failure and disease progression [17].

Previously, we reduced the formation of drug-induced aneuploid/polyploid (DIAP) cells and increased cell death with a combination alisertib plus ibrutinib (BTK inhibitor) plus rituximab (anti-CD20 antibody) [18]. Here, we investigated the molecular and cellular processes and signaling pathways of therapy failure due to DIAP in DH/DE-DLBCL and to discover novel therapeutic targets. We describe a procedure to isolate DIAP cells of alisertib treated DH/DE-DLBCL cell lines by fluorescence-activated cell sorting (FACS) using histone 2B-GFP transfected cells. Time-lapse imaging of single viable polyploid cells demonstrated that DIAP cells divide by reductive cell divisions in the absence of drug. FACS-isolated DIAP cells were subjected to RNA isolation for genomics, proteomics and kinome profiling. We then evaluated differential gene and protein expression by means of gene ontology (GO), biological pathway (Kyoto Encyclopedia for Genes and Genomes (KEGG)) and BIOCARTA systems for therapeutic target discovery. We identified 8 proteins that are significantly up-regulated, and 6 proteins that are significantly down-regulated in DIAP cells. Several of these proteins are involved in cell cycle processes including mitotic cell cycle regulation. Most notable are the up-regulated RANGAP1, TPX2 and KPNA2 proteins, which are associated with the mitotic spindle assembly checkpoint regulation and are likely to be responsible for induction of aneuploidy/polyploidy of daughter cells with resultant therapy failure. Collectively our data provide a mechanism to target DIAP cells by developing novel drug combinations that can be evaluated in clinical trials in high grade DLBCL.

## RESULTS

### DIAP cells undergo reductive cell divisions: a potential mechanism for therapy failure

**Figure 1:**
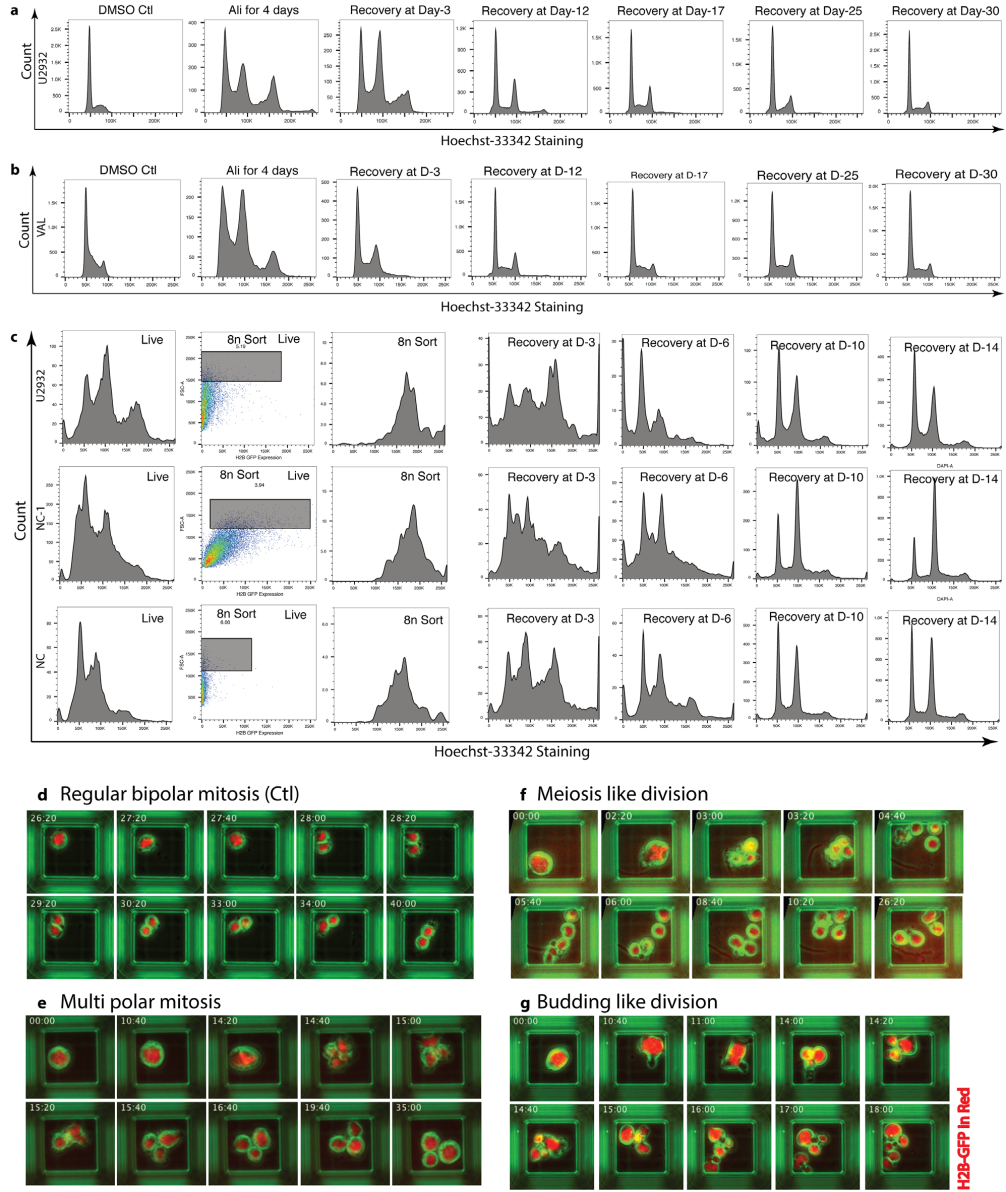
DIAP cells are capable of undergoing reductive cell divisions **(a** & **b)** Cell cycle analyses of U2932 & VAL cells respectively after treatment with 50nM alisertib for 4 days followed by recovery in the absence of drug up to 30-days shows that DLBCL cells establish a diploid 2n-near aneuploid population. **(c)** Polyploidy was induced by treating cells with 50nM alisertib for 4 days, then 8n-cells were sorted based on H2B-GFP expression and utilized forward scatter plot which correspond to cell size by FACS sorting followed by recovery of 8n-cells. Cell cycle analysis showed that with time 8n polyploid cells can shift back to 2n-near aneuploid cells. **(e, f, g)** Time-lapse single cell imaging of 8n cells (same as **c**), showed that polyploid cells can divide into aneuploid cells by multipolar mitosis, meiosis-like or budding-like cell division respectively compared to DMSO treated control cells by binary cell division **(d)**.

Previously we demonstrated that combining alisertib (AK-A inhibitor) with ibrutinib (BTK inhibitor) plus rituximab partially abrogates the formation of alisertib-induced aneuploid/polyploid cells by inhibiting the ERK/MAPK and PI3K/AKT/mTOR signaling pathways [18]. We hypothesized that during the absence of treatment (off-therapy period) DIAP cells could undergo reductive cell divisions. We demonstrated that during 4-days of alisertib treatment, DH/DE-DLBCL cells become polyploid (≤4n). When these polyploid cells are allowed to grow in the absence of alisertib they revert back to 2n-near aneuploidy, and by day-30 there is a complete absence of these polyploid cells (Figure 1a, 1b). Loss of polyploid cells could be due to cell death, reductive cell divisions, or a combination of both respectively.

To determine if reductive mitoses play a role, we enriched H2B-GFP transfected 8n polyploid cells (DIAP) by FACS sorting (Figure 1c) and cultured these cells in the absence of drug. Within 3 days, the number of polyploid cells decreased substantially, coinciding with an increase in 2n-near aneuploid cells. By day-14, the 2n-near aneuploid cells were the predominant population. Together these experiments suggested that DIAP cells are able to revert to 2n-near aneuploid cells in the absence of drug.

Polyploid cells are known to reduce ploidy through several reductive cell division phenomena i.e. multipolar mitosis, meiotic division, and budding-like division. To determine whether one or more these mechanism are responsible for the conversion of polyploid cells to 2n-near aneuploid cells, we conducted time-lapse real-time single cell imaging using FACS sorted enriched 8n-polyploid cells. We plated 8n-polyploid cells in 50 μm microwells to facilitate single cell imaging at 20-minute intervals and tracking these cells over a period of 48 hours. DMSO treated 2n cells were used as a control. Time lapse images of single cells revealed that DIAP cells, which are larger in size than DMSO treated control cells, divide in the absence of drug either via multipolar mitosis (Figure 1e), meiosis-like cell division (Figure 1f) or budding-off daughter cells (Figure 1g). This is in contrast to DMSO treated cells which divide by normal binary division (Figure 1d). Together these data suggest that DIAP cells can undergo reductive cell division(s) by multiple different mechanism and may be responsible for therapy failure.

### Enrichment of DIAP cells for genomic and proteomic analysis

**Figure 2:**
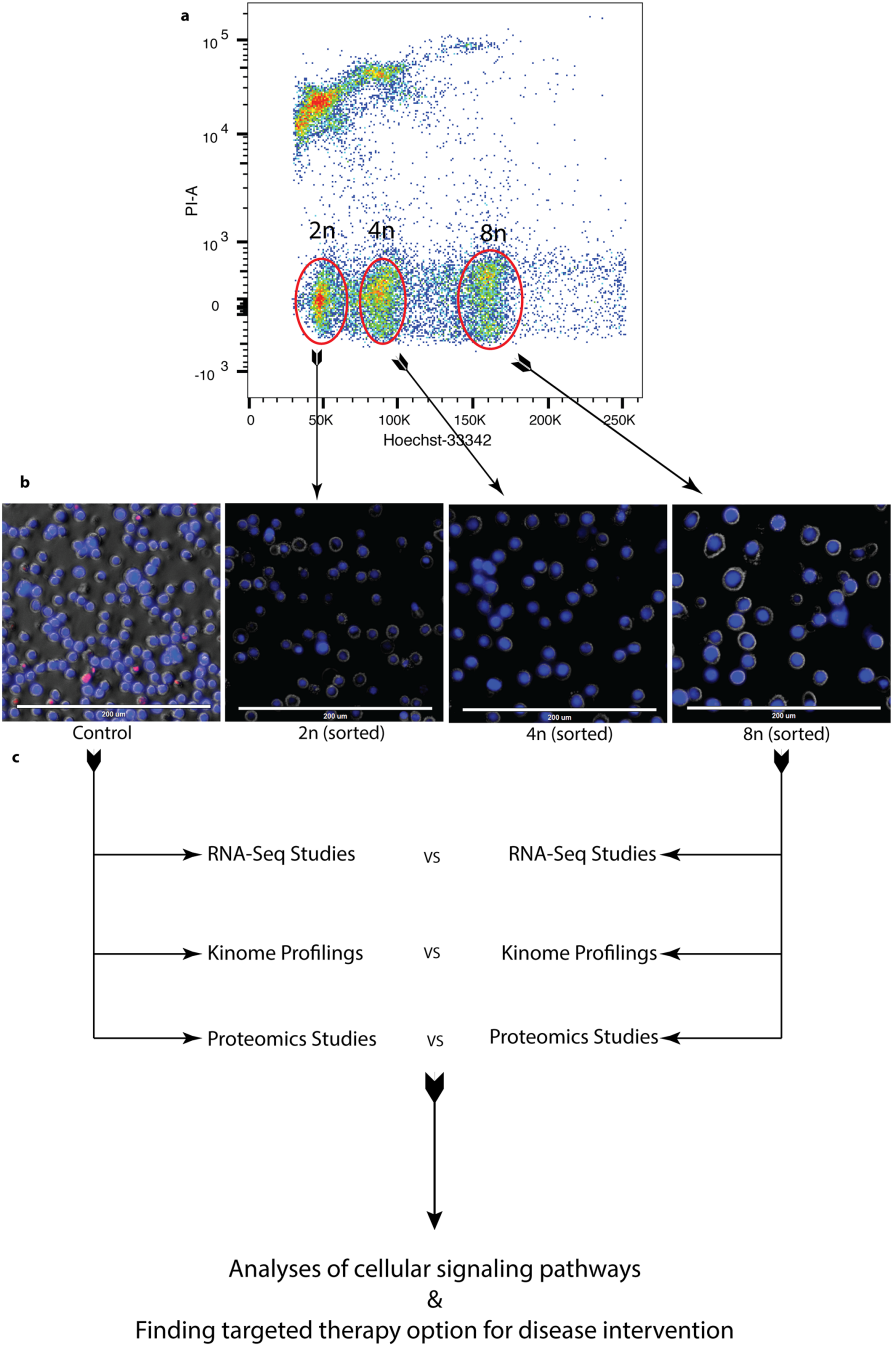
Enrichment of DIAP cells for genomic and proteomic analyses **(a)** Flow data shows different populations of cells after treatment sorted using FACS with Hoechst-33342 staining. **(b)** Images of sorted cells. **(c)** Sample preparation and analysis workflow for RNA-Seq, Proteomic, and Kinome profiling.

In order to determine the pathways that are required for DIAP cells survival, DLBCL cells were treated with 50nM alisertib for 4-days to induce polyploidy, stained with Hoechst-33342 and FACS sorted for live 2n-near aneuploid, 4n and 8n cells (Figure 2a). Images of respective cell populations after sorting are shown (Figure 2b). These sorted cell populations are utilized for molecular analyses with their corresponding DMSO control population (Figure 2c).

### DIAP cells up-regulate genes involved in DNA repair, replication and immune response

**Figure 3:**
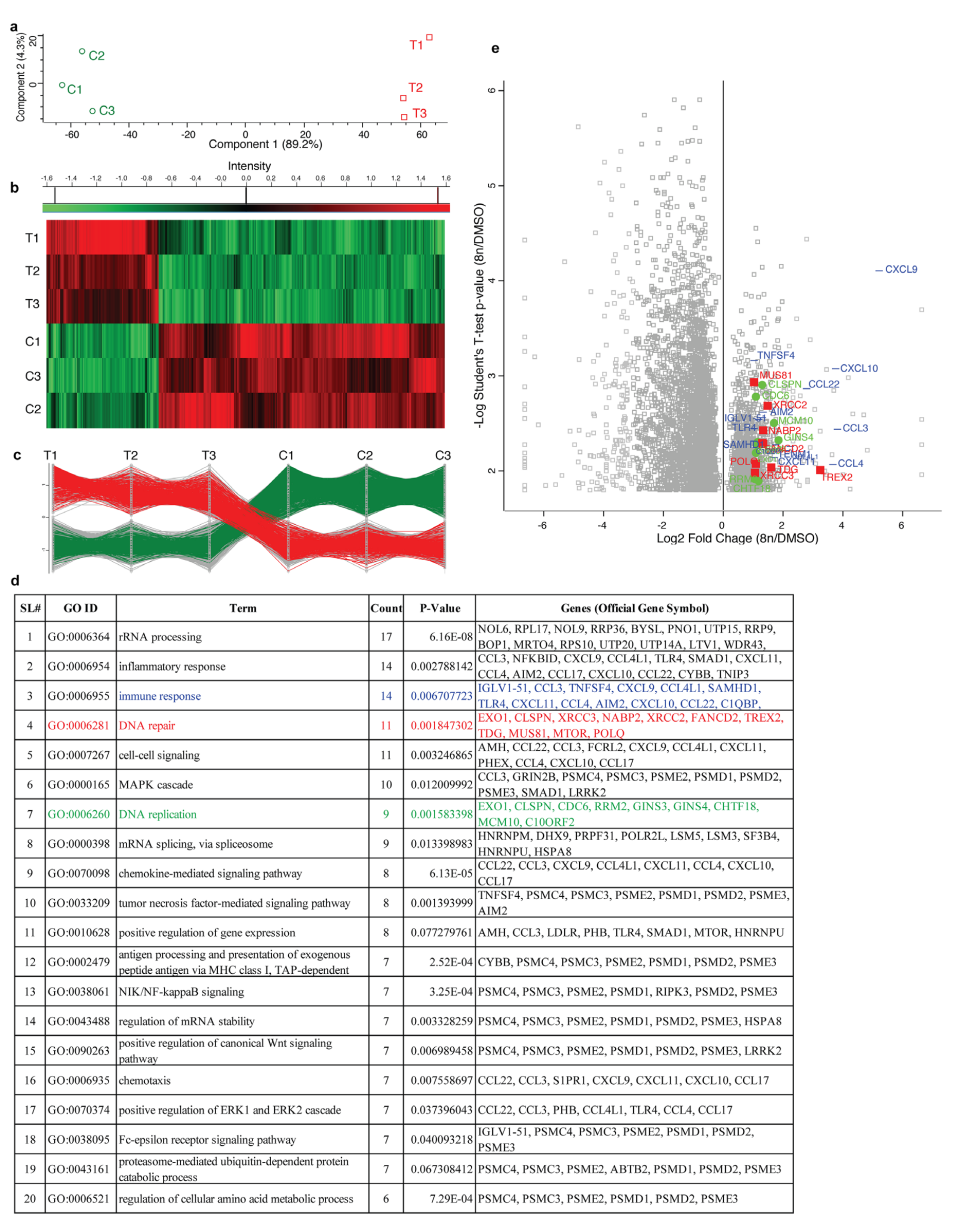
RNA sequencing of DIAP cells shows up-regulation of genes involved in DNA repair, DNA replication and immune response **(a)** PCA (Principal component analysis) of control vs treated (8n) biological replicates. **(b)** Gene expression heat map showed that 1,234 genes were up-regulated, and 3,186 genes were down-regulated in all three biologic replicates in treatments vs. controls. **(c)** Profile plot showed that 404 up-regulated and 605 down-regulated genes follow the same pattern across treatment vs control in all biological replicates. **(d)** DAVID database search with up-regulated genes identify GO terms associated with DNA repair, DNA replication, immune response along with others. **(e)** Log2 fold change vs -log10 P-value visualization of genes associated with DNA repairs (red), DNA replication (green) and immune response (blue). All right most (same vertical line) genes were identified only in 8n-cells but not in DMSO control. On the other hand, all left most (same vertical line) genes were identified only in the DMSO control but absent in 8n-cells.

For RNA-Seq analysis, U2932 cells were treated with 50nM alisertib and DMSO as a control. After 4 days of treatment 8n cells were FACS sorted then RNA isolated from both 8n cells and DMSO treated control sample (Figure 2). All experiments were conducted with three biological replicates to infer statistical significance for differential gene expression [19]. A total of 63,677 transcripts were identified that passed all quality control tests. T-tests demonstrated that 4,420 genes were significantly (p≤0.05) differentially expressed when alisertib treated 8n cells are compared to control (DMSO). Principal component analysis (PCA) indicated that the first principal component accounted for >80% of the variance in gene expression data between treatment and control groups (Figure 3a). Quality clustering analysis performed after calculating the Z-score (Figure 3b) which found 1,234 genes were up-regulated, and 3,186 genes were down-regulated in treatments vs. controls. Profile plots for gene expression patterns identified 404 genes from the up-regulated set that follow the same pattern, whereas 605 genes follow the same pattern for down-regulated genes (Figure 3c).

We used the ‘DAVID’ Bioinformatics Database to identify GO terms associated only with up-regulated genes. GO term related to DNA repair (GO:0006281, P = 0.001847302, Count = 11, Red color), DNA replication (GO:0006260, P = 0.001583398, Count = 9, Green color) and Immune response (GO:0006955, P = 0.006707723, Count = 14, Blue color), were identified along with other GO terms (Figure 3d). To identify polyploidy specific proteins responsible for DNA repair, DNA replication and immune response, Log2 fold change and -Log10 P-value were co-visualized in a volcano plot for each gene using DIAP cells compared to DMSO control cells (Figure 3e). Of the 4,420 genes with significantly different transcript abundance, 1,200 are significantly up-regulated, and 3,072 are significantly down-regulated. Of the up-regulated genes, 11 genes are involved in DNA repair (*EXO1, CLSPN, XRCC3, NABP2, XRCC2, FANCD2, TREX2, TDG, MUS81, MTOR, POLQ*), all of which showed a ≥2-fold increase in expression (shown in red squares). A total of 9 genes are involved with DNA replication (*EXO1, CLSPN, CDC6, RRM2, GINS3, GINS4, CHTF18, MCM10, C10ORF2*), all of which showed a ≥2-fold increase in expression in DIAP (shown in green circles). Similarly, 14 genes involved with immune response (*IGLV1-51, CCL3, TNFSF4, CXCL9, CCL4L1, SAMHD1, TLR4, CXCL11, CCL4, AIM2, CXCL10, CCL22, C1QBP, TENM1*) exhibited a ≥2-fold increase in expression in DIAP cells (shown in blue lines).

### DIAP cells regulate signaling pathways of cell proliferation and survival

The Protein Tyrosine Kinase (PTK) PamChip assay measures the phosphorylation of 196 peptides, representing the kinase activity in each sample (4-days alisertib treated 8n and untreated controls, N=3). The Log_2_ signal intensities depict the phosphorylation signals per peptide (x), and significant effects resulting from an Anova-Dunnet’s test between the treatment populations (alisertib 50nM, 4 days) and DMSO control were measured. 41 peptides were significantly (p≤0.05) increased in phosphorylation of alisertib treated (50nM, 4 days) 8n cells vs. control, of these the top 20 peptides are listed (Table 1a). The DAVID Bioinformatics Database search of these 20 kinase substrates significantly enriched GO terms related to TK phosphorylation cascades and signal transduction. More specifically GO term related to MAPK cascade (GO:0000165, P = 4.72E-06, Count = 6), signal transduction (GO:0007165, P =0.00480611, Count = 6), positive regulation of PI3K signaling (GO:0014068, P = 4.69E-07, Count = 5) are identified with other related GO IDs (Table 1b). Similarly, KEGG analysis identified 31 biological pathways related to tyrosine kinase signaling of which the top ten are shown (Table 1c).

Table 1Kinome profile of DIAP cells that regulate signaling pathways of cell proliferation and survival

**1a:** List of top 20 peptides kinases that are significantly up-regulated in 8n cells vs. control

SL#	Uniprot Accession	Protein-name	Peptide Sequence	Log2 FC_(8n/Ctl)	-log10 P-Value
1	Q13164	mitogen-activated protein kinase 7(MAPK7)	AEHQYFMTEYV A T	1.49	2.30
2	Q08999	RB transcriptional corepressor like 2(RBL2)	VPTVSKGTVEGNY	1.34	2.91
3	Q15303	erb-b2 receptor tyrosine kinase 4(ERBB4)	QALDNPEYHNASN	1.29	2.51
4	P46108	CRK proto-oncogene, adaptor protein(CRK)	GPPEPGPYAQPSV	1.20	1.96
5	P07355	annexin A2(ANXA2)	HSTPPSAYGSVKA	1.15	3.11
6	P06241	FYN proto-oncogene, Src family tyrosine kinase(FYN)	TA TEPQYQPGENL	1.15	1.83
7	P17948	fms related tyrosine kinase 1(FLT1)	DFGLARDIYKNPD	1.14	3.47
8	P27361	mitogen-activated protein kinase 3(MAPK3)	GFLTEYV A TR	1.14	2.66
9	P11802	cyclin dependent kinase 4(CDK4)	EIGVGAYGTVYKA	1.07	2.69
10	P09619	platelet derived growth factor receptor beta(PDGFRB)	PNEGDNDYIIPLPDP	0.99	1.86
11	P54762	EPH receptor B1(EPHB1)	DDTSDPTYTSSLG	0.98	3.38
12	P06401	progesterone receptor(PGR)	EQRMKESSFYSLC	0.98	1.64
13	P53778	mitogen-activated protein kinase 12(MAPK12)	SEBTGYVVTR	0.97	1.43
14	P20963	CD247 molecule(CD247)	KDKMAEAYSEIGM	0.96	3.02
15	Q14765	signal transducer and activator of transcription 4(STAT4)	PSDLLPMSPSVY A	0.96	2.18
16	Q15375	EPH receptor A7(EPHA7)	TYIDPETYEDPNR	0.95	4.88
17	Q02763		SRGQEVYVKKTMG	0.93	1.34
18	P22681	Cbl proto-oncogene(CBL)	EGEEDTEYMTPSS	0.93	1.43
19	P20963	CD247 molecule(CD247)	DKMAEAYSEIGMK	0.92	2.32
20	P07332	FES proto-oncogene, tyrosine kinase(FES)	REEADGVYAASGG	0.91	3.61

**1b:** Top 20 GO term associated with significantly up regulated kinases after searching in DAVID database

SL#	GO ID	Term	Count	P-Value	Genes (Uniprot Accession)
1	GO:0018108	peptidyl-tyrosine phosphorylation	7	5.91E-09	P54762, P07332, P09619, P06241, P17948, Q15303, Q15375
2	GO:0000165	MAPK cascade	6	4.72E-06	P09619, P06241, P27361, P53778, Q13164, Q15303
3	GO:0007165	signal transduction	6	0.00480611	P06401, P09619, P11802, P53778, Q13164, Q15303
4	GO:0006351	transcription, DNA-templated	6	0.0397312	P06401, Q08999, Q14765, P27361, P53778, Q15303
5	GO:0014068	positive regulation of phosphatidylinositol 3-kinase signaling	5	4.69E-07	P09619, P22681, P06241, P17948, Q15303
6	GO:0046777	protein autophosphorylation	5	2.28E-05	P54762, P07332, P09619, P17948, Q15303
7	GO:0038083	peptidyl-tyrosine autophosphorylation	4	8.32E-06	P07332, P06241, P27361, Q15303
8	GO:0048010	vascular endothelial growth factor receptor signaling pathway	4	4.92E-05	P06241, P53778, P17948, P46108
9	GO:0014066	regulation of phosphatidylinositol 3-kinase signaling	4	6.26E-05	P09619, P06241, P27361, Q15303
10	GO:0048013	ephrin receptor signaling pathway	4	8.37E-05	P54762, P06241, Q15375, P46108
11	GO:0001934	positive regulation of protein phosphorylation	4	2.66E-04	P07355, P27361, Q15303, Q15375
12	GO:0038096	Fc-gamma receptor signaling pathway involved in phagocytosis	4	2.66E-04	P20963, P06241, P27361, P46108
13	GO:0016477	cell migration	4	6.46E-04	P09619, P06241, P17948, Q15303
14	GO:0043066	negative regulation of apoptotic process	4	0.01012794	P09619, P22681, Q13164, Q15303
15	GO:0006468	protein phosphorylation	4	0.01018863	P07332, P11802, P06241, P27361
16	GO:0008284	positive regulation of cell proliferation	4	0.01080736	P09619, P11802, P17948, Q15303
17	GO:0045944	positive regulation of transcription from RNA polymerase II promoter	4	0.0732784	P06401, Q14765, P27361, Q13164
18	GO:0036120	cellular response to platelet-derived growth factor stimulus	3	1.46E-04	P09619, P22681, P06241
19	GO:0043552	positive regulation of phosphatidylinositol 3-kinase activity	3	4.41E-04	P09619, P17948, Q15303
20	GO:0048146	positive regulation of fibroblast proliferation	3	0.0013384	P07355, P09619, P11802

**1c:** Top 10 tyrosine kinase signaling pathways identified by KEGG analysis

SL#	KEGG ID	Term	Count	P-Value	Genes (Uniprot Accession)
1	hsa04660	T cell receptor signaling pathway	6	2.56E-06	P20963, P22681, P11802, P06241, P27361, P53778
2	hsa04360	Axon guidance	5	1.67E-04	P54762, P07332, P06241, P27361, Q15375
3	hsa04510	Focal adhesion	5	0.00105318	P09619, P06241, P27361, P17948, P46108
4	hsa04015	Rap1 signaling pathway	5	0.00113164	P09619, P27361, P53778, P17948, P46108
5	hsa04010	MAPK signaling pathway	5	0.00232085	P09619, P27361, P53778, Q13164, P46108
6	hsa04151	PI3K-Akt signaling pathway	5	0.00688865	Q08999, P09619, P11802, P27361, P17948
7	hsa05200	Pathways in cancer	5	0.01085798	P09619, P22681, P11802, P27361, P46108
8	hsa05220	Chronic myeloid leukemia	4	5.51E-04	P22681, P11802, P27361, P46108
9	hsa04012	ErbB signaling pathway	4	9.59E-04	P22681, P27361, Q15303, P46108
10	hsa04722	Neurotrophin signaling pathway	4	0.0024249	P27361, P53778, Q13164, P46108

### Anti-apoptosis and cell proliferation in DIAP cells are a mechanism of therapy resistance

**Figure 4:**
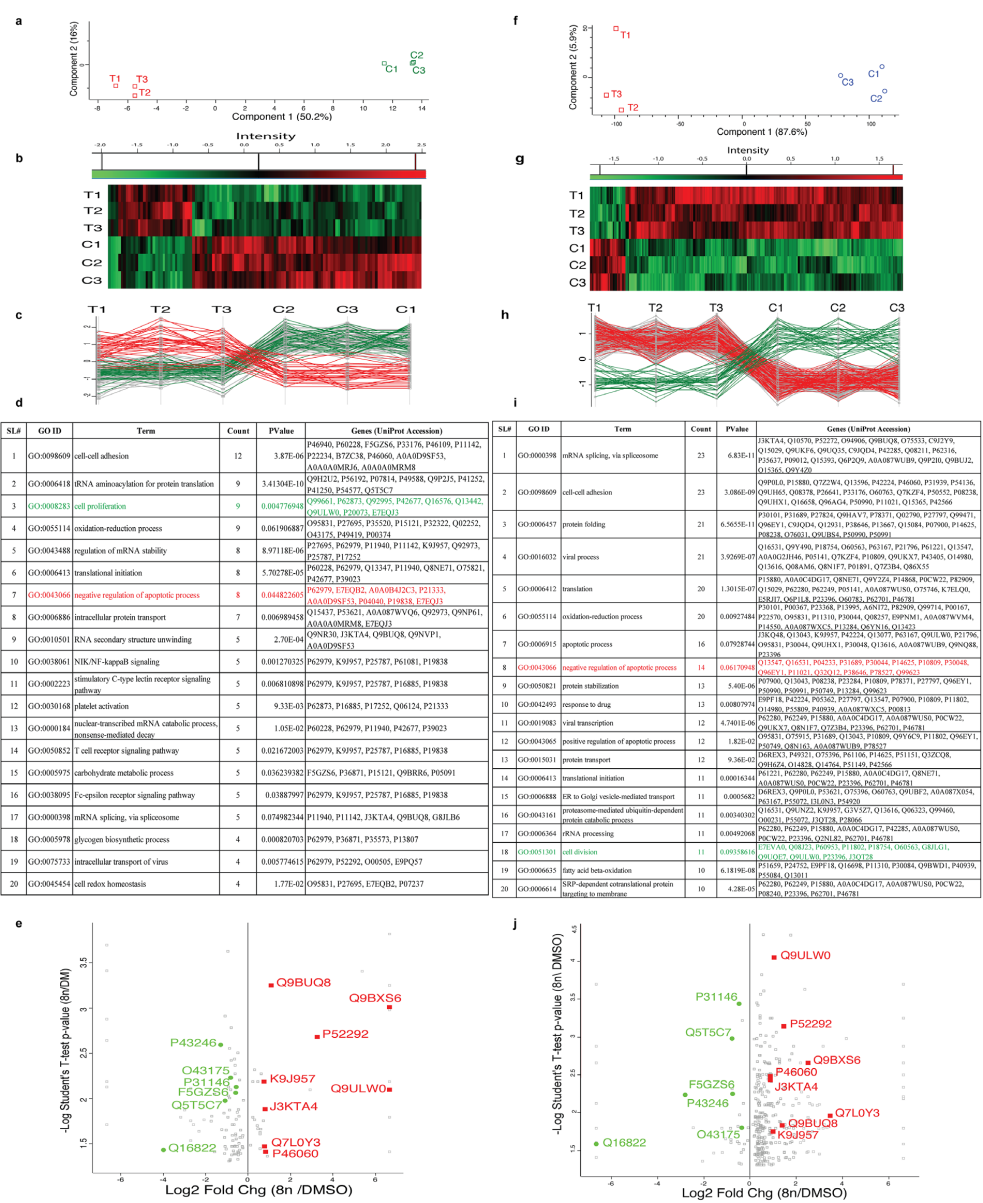
Anti-apoptosis and cell proliferation in DIAP cells are a mechanism of therapy failure Left panel **(a, b, c, d** & **e)** represents results from U2932 cells and right panel **(f, g, h, i & j)** represents results from VAL cells. **(a & f)**. Principal component analysis (PCA) showed concordance among biological replicates and variance between control vs. treatment groups. **(b & g)**. Heat map of protein expression treatment vs control. **(c & h)**. Profile plot protein expression pattern respectively for both cell lines. **(d & i)**. Top 20 enriched GO terms including negative regulation of apoptosis and positive regulation for cell proliferation respectively for U2932 and VAL cell lines. **(e & j)**. Volcano plot shows 8 proteins are significantly up-regulated and 6 proteins are down-regulated in both cell lines. All right most (same vertical line) proteins identified only in 8n-cells but not in DMSO control. On the other hand, all left most (same vertical line) proteins identified only in DMSO control but absent in 8n-cells.

To confirm the findings from RNA-Seq and kinome profiling, we performed proteomic studies on U2932 (DE-DLBCL) and VAL (DH-DLBCL) cell lines. In total 2,225 and 2,149 proteins were identified that passed a minimum of two peptides identified at 0.1% peptide FDR and 90-99.9% protein confidence by the Protein Profit algorithm, within Scaffold respectively for U2932 and VAL cell lines. Of these, 127 and 404 proteins were significantly (p≤0.05) differentially expressed in DIAP vs. control respectively in U2932 and VAL cells. Principal component analysis (PCA) showed concordance among biological replicates and indicated the greatest variance between control vs. treatment groups (Figure 4a and 4f). To further check experimental data quality, clustering was performed after calculation of the Z-score (Figure 4b and 4g) respectively, for U2932 and VAL cells. We found that 34 and 359 proteins were up-regulated and 93 and 45 proteins were down-regulated, respectively, in the treatment vs. control groups. A profile plot showed protein expression patterns that identified 34 and 79 proteins as up-regulated that follow the same pattern, respectively, and 35 and 44 proteins follow the same pattern as down-regulated, respectively (Figure 4c and Figure 4h).

Searching for significantly different proteins from both cell lines utilizing the DAVID bioinformatics database showed further consistency in enriched GO terms. DIAP cell populations in both cell lines enrich for negative regulation of apoptosis (GO:0043066, P = 0.044822605, Count = 8, Red color), (GO:0043066, P = 0.06170948, Count = 14, Red color) and positive regulation for cell proliferation (GO:0008288, P = 0.004776948, Count = 9, Green color), (GO:0051301, P = 0.09358616, Count = 11, Green color), respectively, for U2932 and VAL DLBCL cells (Figure 4d and Figure 4i). Volcano plots that co-visualize Log2 fold changes (DIAP/DMSO) and -Log10 P-Value for each protein revealed that 8 proteins (Q9BXS6, Q9ULW0, P52292, Q9BUQ8, P46060, J3KTA4, Q7L0Y3, K9J957) were significantly up-regulated in both cell lines and 6 proteins (P31146, F5GZS6, O43175, Q5T5C7, P43246, Q16822) were down-regulated in both cell lines (Figure 4e and Figure 4j) shown in red and green, respectively.

### DIAP cells dysregulate the mitotic spindle assembly checkpoint to facilitate therapy failure

**Figure 5:**
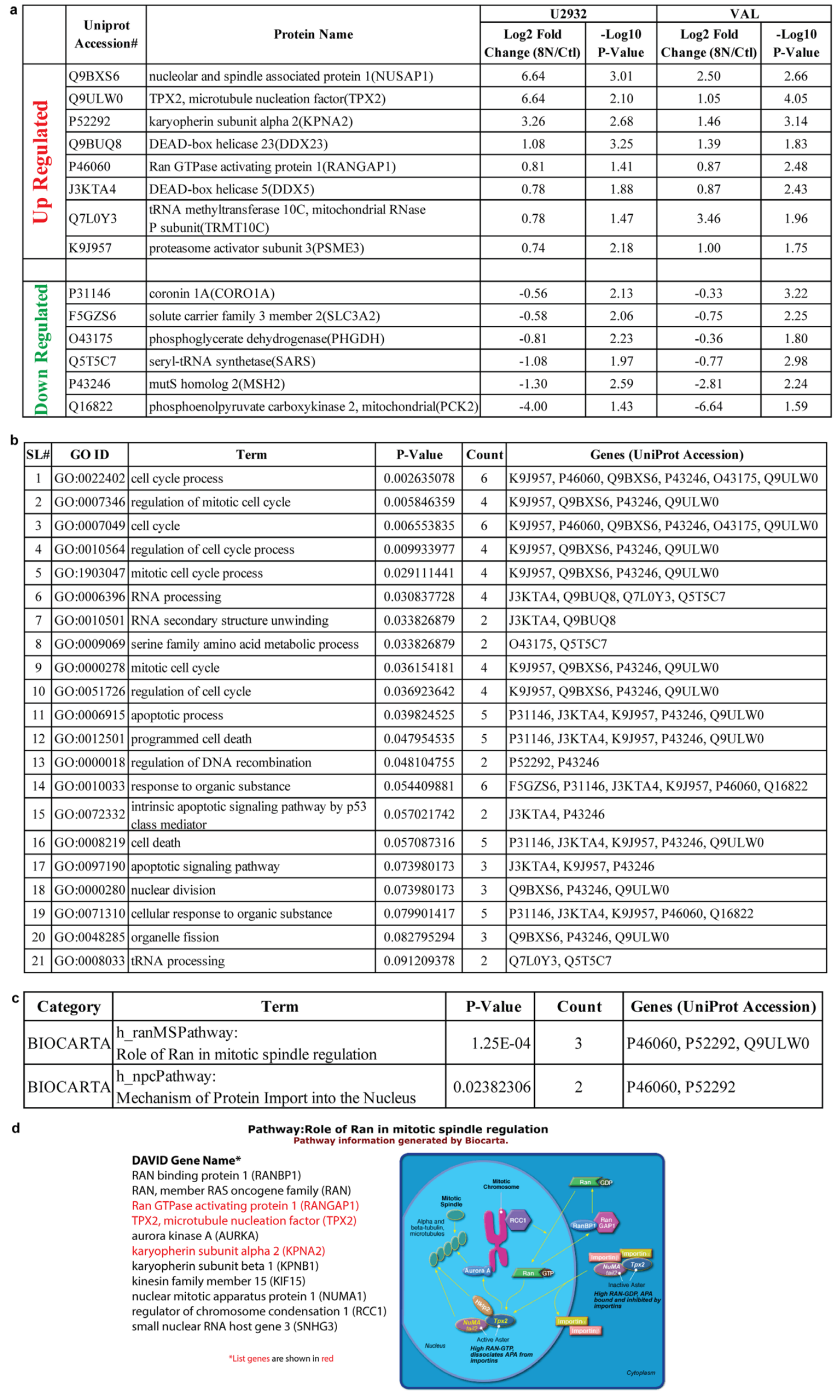
DIAP cells dysregulate the mitotic spindle assembly checkpoint with over-expression of KPNA2, RAN-GAP1 and TPX2 to facilitate therapy failure **(a)** Common up-regulated and down-regulated proteins in U2932 and VAL DIAP cells. **(b)** GO terms associated with common up-regulated and down-regulated proteins in the DAVID database. **(c** & **d)** BIOCARTA pathway analysis shows associated signaling pathway and proteins.

To investigate the role of the 8 dysregulated proteins that are significantly up-regulated and 6 proteins that are significantly down-regulated in both U2932 and VAL cell lines (Figure 5a), we searched the DAVID bioinformatics database. We discovered that these proteins are closely involved in the mitotic phase of the cell cycle in the identified GO terms (Figure 5b). Mining of the BIOCARTA pathways revealed that the most altered biological pathway was *h_ranMSPathway*: *role of Ran in mitotic spindle regulation* with a P = 1.25E-04 (Figure 5c and Figure 5d). Therefore, dysregulated spindle assembly checkpoint may contribute to therapy failure, but we need to know the underlying protein network.

### Interactions among Myc, Bcl2 with KPNA2, Ran-GAP1, TPX2 and AK-A in DIAP cells expedite disease relapse

**Figure 6:**
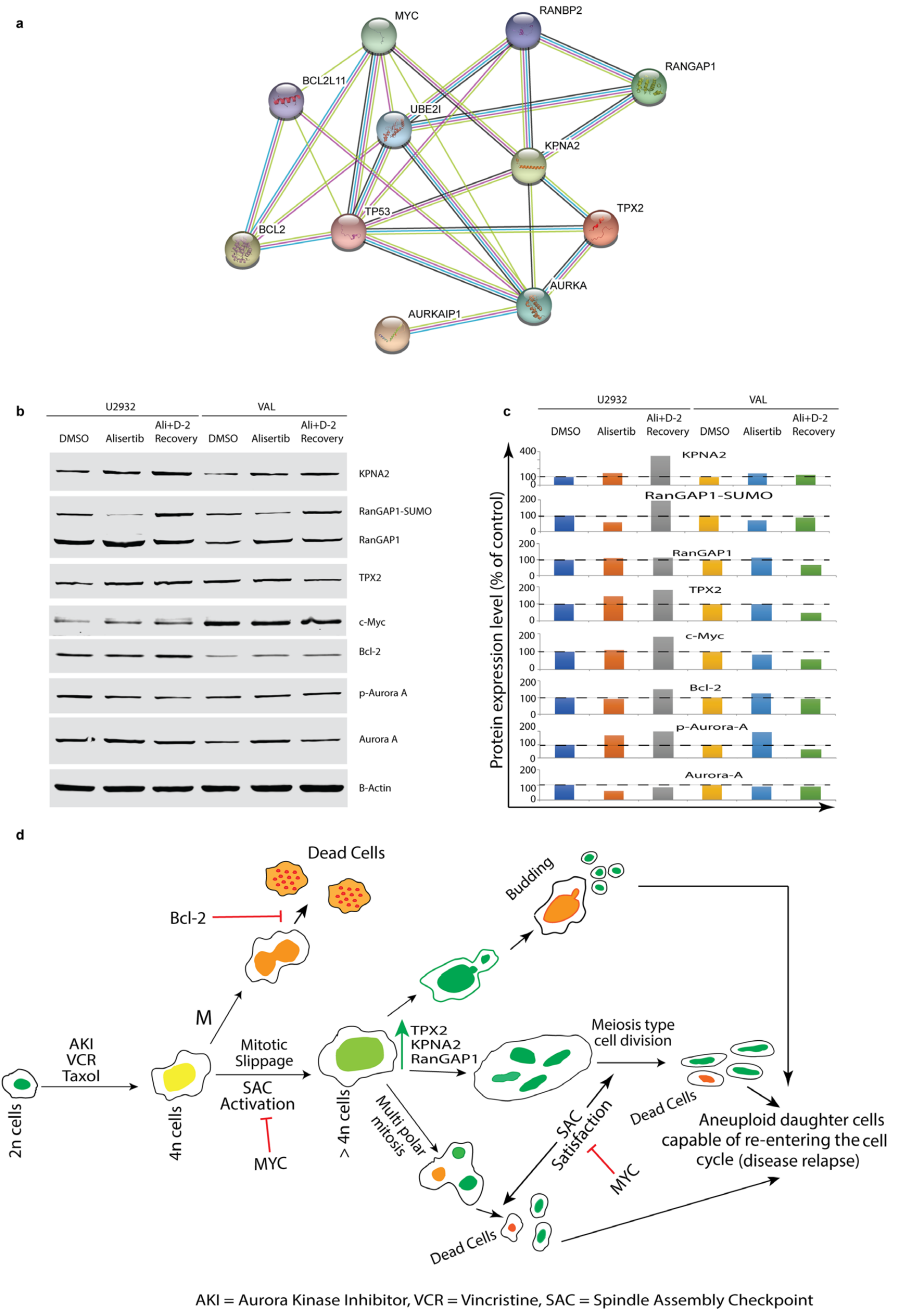
Interactions among Myc, Bcl2 with KPNA2, Ran-GAP1, TPX2 and AK-A in DIAP cells expedite disease relapse **(a)** STRING database shows interactions among up-regulated proteins with AK-A, Myc and Bcl2. **(b** & **c)** Western blotting confirmed up-regulated target proteins and their quantification after normalization. **(d)** Role of KPNA2, RanGAP1 and TPX2 in reductive cell division(s) in DIAP cells represented along with Myc, Bcl2 and AK.

Protein network analysis using the STRING database confirmed the interactions among KPNA2, Ran-GAP1 and TPX2 with AK-A, Bcl2 and Myc expression (Figure 6a). Western blotting analysis of U2932 and VAL cells treated with alisertib for 4 days and followed by 2-days recovery from treatment confirm these proteins are up-regulated in DIAP cells (Figure 6b and 6c). We surmise that these up-regulated proteins may provide DIAP cells with the ability to divide either by multipolar mitosis, meiosis-like division, or budding type division (Figure 6d). Discovery of Ran signaling in DIAP cells suggests opportunities for targeting this pathway in combination with an AK inhibitor (e.g. alisertib) to prevent or disrupt polyploidy. In addition, further studies are warranted investigating anti-DLBCL chemotherapies that induce DIAP to identify common and unique mechanism of therapy failure, target identification and novel therapies to overcome drug resistance.

## DISCUSSION

MYC tightly regulates AK activity and in collaboration with BCL2 promotes an anti-apoptotic response to cell cycle inhibitors in DH/DE-DLBCL. We investigated AK inhibition induced aneuploidy/polyploidy in DH/DE-DLBCL to decipher cellular processes, signaling pathways and to identify novel drug targets to disrupt and/or prevent DIAP. Alisertib, an AK-A inhibitor induces polyploidy in DH/DE-DLBCL cells, however when alisertib is removed, DIAP cells undergo reductive cell division to 2n-near aneuploid cells that could re-enter the cell cycle. In addition, the rate of 2n-near aneuploid cell recovery in the absence of drug is faster with VAL cells (*TP53* wild type) compared with U2932 cells (*TP53* mutant), which may be due to a distinct TP53 transcriptional program. H2B-GFP transfected U2932 cells treated with alisertib and enriched for 8n cells (∼80-85% of the total cell population)by FACS sorting followed by time-lapse single cell imaging demonstrated 3 types of reductive cell division in the absence of drug: multipolar mitosis, meiosis-like nuclear fission, and budding of daughter cells. It is surmised that these types of reductive cell divisions occur *in vivo* within tumors exposed to polyploidy inducing drugs and is an escape mechanism leading to disease relapse.

RNA-Seq demonstrated that U2932 cells up-regulate 1,234 genes and down-regulate 3,186 genes indicating a selective growth advantage to DIAP cells by optimizing cell fate processes, cell survival and slowing down of non-essential signaling pathways advantageous for tumor survival. Many of the up-regulated genes are involved in DNA repair, DNA replication and an immune response signature indicating DIAP cells are capable of tolerating genomic instability, promote immune suppression, and the ability to evade immune-mediated cytotoxicity. Kinome profiling of U2932 cells demonstrated a significant differential kinome activation between DMSO control vs. alisertib treated 8n cells. The top 20 kinases that are significantly up-regulated in 8n cells vs. control are MAPK, PI3K, and immune signaling which were similar to that observed with RNA-Seq findings.

Proteomic analysis of VAL and U2932 DIAP cells enriched demonstrated negative regulation of apoptosis and positive regulation for cell proliferation promoting rapid tumor evolution. Volcano plots that co-visualize Log2 fold changes and -Log10 P-value for each protein revealed that 8 proteins were significantly up-regulated and 6 proteins were down-regulated in both cell lines. Both RNA-Seq and proteomics enriched for dysregulation of the mitotic spindle assembly checkpoint regulators RAN-GAP1, TPX2, and KPNA2, which we propose is a mechanism of alisertib-induced polyploidy. MYC over-expression induces chromosome missegregation, which impacts microtubule nucleation, aster coalescence and centrosome dynamics. Continued MYC expression in the presence or absence of alisertib most likely contributes to spindle abnormalities. In addition, deregulated MYC expression depends on TPX2 over-expression to complete mitosis [20]. Our results demonstrate an additional level of complexity in which RAN (GTP/GDP) signaling propagates chromosomal congression defects leading to polyploidy.

Protein analysis by Western blotting of U2932 and VAL cells treated with alisertib and off-therapy for 2-days confirmed RNA-Seq and proteomics findings. MYC regulated RanGAP, TPX2, and KPNA2 are up-regulated on day 2 of recovering cells with intact Myc and Bcl2 expression, indicating that the spindle assembly checkpoint is dysregulated during induction of polyploidy and these cells are able to escape cell death. In addition, as demonstrated by real-time single cell imaging, day-2 of recovering cells undergo reductive cell divisions (multipolar mitosis, meiosis-like or budding type division) confirming a defective spindle assembly checkpoint as a mechanism of alisertib induced polyploidy. Further, the differential recovery from alisertib observed between U2932 and VAL indicate MYC/BCL2 translocated (DH) have a more aggressive phenotype compared with MYC/BCL2 over-expressed (DE) DLBCL, the former with a worse prognosis [21].

Identification of the RanGTP signaling pathway in DIAP cells provides opportunities for targeting this pathway in combination with AK inhibitors (e.g. alisertib, barasertib) to disrupt drug-induced aneuploidy/polyploidy. In addition, kinome profiling also provides novel targets for therapeutic intervention including immune suppression. Finally, studies are warranted investigating common anti-DLBCL drugs that induce DIAP to disrupt aneuploidy/polyploidy to achieve better outcomes for patients with high grade DLBCL.

## MATERIALS AND METHODS

### Cells and reagents

DH/DE-DLBCL cell lines U2932, U2904 and OCI-Ly18 were obtained from Deutsche Sammlung von Mikroorganismen und Zellkulturen GmbH (DSMZ). VAL cell line was obtained from Dr. Samantha Kendrick, Biochemistry and Molecular Biology, University of Arkansas for Medical Sciences. All cell line authentications were performed using Promega PowerPlex16HS Assay at the University of Arizona Genetics Core. All cell lines were tested for mycoplasma contamination using MycoAlert Mycoplasma Detection Kit (Lonza). U2932 (obtained in 2012), VAL (obtained in 2013), OCI-Ly18 (obtained in 2016), and U2904 (obtained in 2016) were cultured in RPMI-1640 medium (Mediatech, VA) supplemented with 10% fetal bovine serum at 37°C in a humidified atmosphere containing 5% CO_2_. Alisertib was purchased (Selleck Chemicals, USA). The compounds were dissolved in 10mM in DMSO as a stock solution and then further diluted to desired concentrations for *in vitro* experiments. NC, NC-1, A6C1 are different clones of U2932 cells expressing H2B-GFP.

### Aneuploidy recovery assay

Flow cytometry-based assays were utilized to quantify polyploidy in live cells based on membrane permeable dye Hoechst-33342 staining. U2932 and VAL cells were treated with alisertib 50nM for 4 days to induce polyploidy then drugs were washed out to recover cells for indicated time period and polyploidy were check in different time point. Stained cells are run in BD-LSR-II and analyzed with FlowJo software (FlowJo, LLC).

### H2B-GFP expression

3^rd^ generation Lentiviral transfer plasmid pHR_dSV40-H2B-GFP (Plasmid #67928) was purchased from addgene and 3^rd^ generation Lenti-vpak packaging kit was bought from Origene (Cat # TR30037P5). HEK293T cells were purchased from ATCC and virus produced as per the Origene protocol. After transfection, GFP expression was used as a marker for FACS sorting on individual cells expressing GFP and later expanded these cells into different clones with differential GFP expression levels (U2932, NC, NC-1, A6C1).

### Polyploidy live cells sorting

In our previous publication [18], we showed that polyploid cells are metabolically active and large in size compared to normal their counterpart. We used cell size (FSC-A) and H2B-GFP expression which is proportional to cells’ DNA content to sort Polyploid live cells [22].

### Time-lapse single cell imaging

Hematopoietic cells are non-adherent cells which are not suitable for live single cell imaging as it is difficult to follow a single cell for certain time point due to their suspension nature and motility. To image a single cell for a specified time period we used specialized Microgrid arrays (MGA-050-02 & MGA-125-02) from microsurfaces Pty Ltd that can hold single cell in individual Microgrid arrays for imaging the same cell for specified time period. After sorting, live polyploid cells were set in the Microgrid array plate following manufacturer specified protocol. Cells were imaged with an in-house Nikon Eclipse Ti-E inverted microscope. The microscope is equipped with automated stage control, a perfect focus, and an Okolab cage incubator to maintain stable humidity, temperature and CO_2_ levels. This set up can image live cells for 6-day periods without a reduction in the growth rate or viability of cells [23]. Images were captured every 20 minutes over a 48-hour period in order to visualize reductive cell division.

### RNA-Seq analyses

RNA Samples were assessed for quality with an Advanced Analytics Fragment Analyzer (High Sensitivity RNA Analysis Kit – Catalog # DNF-491 / User Guide DNF-491-2014AUG13) and quantity with a Qubit RNA quantification kit (Qubit® RNA HS Assay KitAssay Kit – Catalog # Q32852). Given satisfactory quality and quantity, samples were used for library builds with the standard Kapa Biosystems mRNA HyperPrep Kit (KapaBiosystems KAPA mRNA HyperPrep Kit – Catalog # KK8540 / KapaBiosystems mRNA HyperPrep Kit TDS KR1352 – v4.17). Upon library build completion, samples were assessed for quality and average fragment size with the Advanced Analytics Fragment Analyzer (High Sensitivity NGS Analysis Kit – Catalog # DNF-486 / User Guide DNF-486-2014MAR10). Quantity was assessed with an Illumina Universal Adaptor-specific qPCR kit from Kapa Biosystems (Kapa Library Quantification kit for Illumina NGS – Catalog # KK4824 / KAPA Library Quantification Technical Guide - AUG2014).

After final library QC was completed, samples were equimolar-pooled and clustered for sequencing on the HiSeq2500 machine. The sequencing run was performed using Illumina HiSeq Rapid SBS v2 chemistry (HiSeq Rapid SBS Kit v2 200 cycles - Catalog # FC-402-4021, HiSeq PE Rapid Cluster Kit v2 - Catalog # PE-402-4002), and data were sent to UAGC Biocomputing Group for further analysis and transmission to the client.

### Kinome profiling

After sorting desired population by FACS, cells are lysed and prepared as per the PamGene protocol (Protocol 1160) and shipped to PamGene on dry ice. With PamGene's Protein Tyrosine Kinase (PTK) PamChip, (86402) (PamGene International B.V., https://www.pamgene.com/en/Tyrosine-PamChip.htm), the activity of protein kinases was measured. Each PTK PamChip array contains 196 peptides immobilized on a porous membrane. The peptide sequences (13 amino acids long) harbor phosphorylation sites derived from literature or computational predictions and are correlated with one or multiple upstream tyrosine kinases. A fluorescently labelled anti-phospho Tyr antibody (PY20) was used to detect the phosphorylation activity of kinases present in the sample and quantified using the BioNavigator 6.3 software (PamGene International B.V.). BioNavigator software 6.3 (PamGene International B.V.) was used to determine signal intensities, peptide QC and preselection (phosphorylation kinetics, or increase in signal order time, in 25% of the arrays analyzed), Log 2 transformation, ANOVA-Dunnett’s testing, and data visualization.

### Proteomic analyses

Cells are lysed with RIPA buffer (CST# 9806) in presence of Protease/Phosphatase Inhibitor Cocktail (CST# 5872) followed by acetone precipitation of proteins and handed over to core facility for LC-MS/MS. Proteins identification by LC-MS/MS were performed following core facility’s standard optimized protocol [24]. Tandem mass spectra were searched against the human fasta protein database from UniprotKB downloaded on October 06, 2015 (69,961 entries), to which additional common contaminant proteins (eg., trypsin; obtained at ftp://ftp.thegpm.org/fasta/cRAP) were appended.

### Data analyses

Most of the data analyses were done by Perseus as per their recommended method [25]. Principal component analysis (PCA) attributes the largest variance to the difference between control and treatment groups and shows that control and treatment groups are distinctively different. Clustering were done after normalization to Z-score using median option. Profile plots of two selected clusters showing distinct behavior with respect to control vs treatment group: increased expression in treatment vs decreased expression in control and vice versa.

### Data sharing statement

The RNA-Seq, kinome profiling and proteomic data are available from corresponding author upon request.
